# Successful Treatment of Eosinophilic Chronic Rhinosinusitis and Secretory Otitis Media in Refractory Asthma With Thymic Stromal Lymphopoietin (TSLP) Receptor Monoclonal Antibody

**DOI:** 10.7759/cureus.47798

**Published:** 2023-10-27

**Authors:** Kayoko Kawashima, Osamu Matsuno, Mika Okuno, Takanari Kawabe, Yukiko Hanada

**Affiliations:** 1 Department of Otorhinolaryngology-Head and Neck Surgery, Osaka Habikino Medical Center, Habikino, JPN; 2 Department of Allergology and Rheumatology, Osaka Habikino Medical Center, Habikino, JPN

**Keywords:** type 2 inflammation, tezepelumab, anti-thymic stromal lymphopoietin, chronic rhinosinusitis without nasal polyp, eosinophilic chronic rhinosinusitis

## Abstract

Eosinophilic chronic rhinosinusitis (ECRS) is a type 2 inflammatory disease that frequently co-occurs with bronchial asthma. The current treatment options for ECRS include endoscopic sinus surgery and oral corticosteroid therapy (OCS). However, recurrence after surgery is common, and OCS therapy may cause side effects. We present the case of a 74-year-old woman with severe asthma, ECRS, and secretory otitis media with possible eosinophilic otitis media, who experienced significant improvement in both conditions after treatment with tezepelumab, an anti-thymic stromal lymphopoietin (TSLP) antibody. Tezepelumab treatment led to a reduction in blood and tissue eosinophil counts. It improved the nasal polyp and computed tomography scores, tympanic and hearing test results, and asthma symptoms without using OCSs. Our findings suggest that tezepelumab may be a promising option for those patients with asthma, ECRS, and secretory otitis media who do not respond well to conventional treatment because upstream of the type 2 inflammation pathway is suppressed. Further to this case report, future studies are required to confirm the long-term efficacy and safety of tezepelumab in treating ECRS and secretory otitis media due to type 2 inflammation.

## Introduction

Eosinophilic chronic rhinosinusitis (ECRS) is a type 2 inflammatory disease characterized by chronic inflammation of the upper airways, intractable nasal polyps with eosinophilic infiltration, and olfactory disturbances [1]. Endoscopic sinus surgery (ESS) and oral corticosteroid (OCS) therapy are the primary treatment modalities for ECRS. Within the patients with type 2 chronic rhinosinusitis with nasal polyps (CRSwNP), there is a group of about 25%-30% of subjects who will relapse after oral glucocorticosteroids (GCSs) or conventional sinus surgery and often need several surgeries in a lifetime. More than 60% of these patients also have late-onset asthma. Patients with CRSwNP who relapse after systemic GCSs and/or adequate surgery and are symptomatic and relevantly impaired in their quality of life (QOL) should be identified as severe and uncontrolled and eventually treated with biologics as an innovative option. It must be remembered that treatment with systemic GCSs or surgery is associated with adverse events, specifically when GCSs are applied repeatedly or for long-term, and possible complications, specifically when surgeries are performed repeatedly. Major complications are reported in about 0.5%-1% of sinus surgeries. Eosinophilic otitis media (EOM) is a type 2 inflammatory disease that presents as a refractory condition characterized by yellow colloidal middle ear effusions and numerous eosinophils [2]. These diseases frequently co-occur with bronchial asthma. Recently, several biological agents, including anti-interleukin (IL)-4 receptor-alpha, anti-immunoglobulin E (IgE), anti-IL-5, anti-IL-5 receptor-alpha, and anti-thymic stromal lymphopoietin (TSLP) antibodies, have been approved for the treatment of severe type 2 asthma. Among these, anti-TSLP antibodies (tezepelumab) effectively suppress the upstream factors that cause type 2 inflammation, with potentially beneficial effects in cases that respond poorly to conventional antibody drugs [3]. IL-4, IL-5 and IL-13 cytokines are key cytokines of type 2 immune reactions. Increased tissue and blood eosinophils, as well as elevated tissue and serum total IgE concentrations, are typical signs of type 2 immune reactions. Biologics targeting these cytokines and IgE have been developed for asthma, atopic dermatitis, and other type 2 diseases. Since CRSwNP in the United States and Europe represents type 2 immune reactions in most patients, these biologics can also be applied to nasal polyp disease. However, in Japan, only anti-IL-4 receptor-alpha antibodies are currently approved for the treatment of CRSwNP. Herein, we present a case of a patient with severe asthma who showed improvement in concomitant ECRS and secretory otitis media after treatment with tezepelumab.

## Case presentation

**Figure 1:**
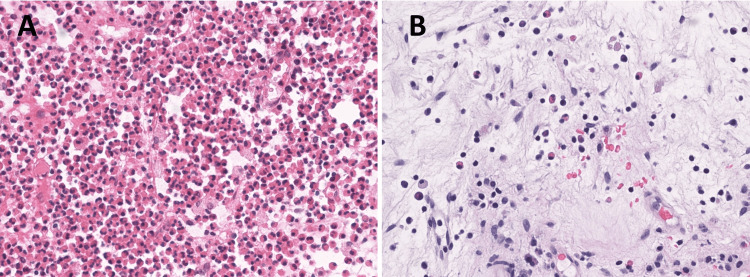
Pathological findings. (A) Initial surgery, (B) Three months after treatment. Compared to the histological findings at the time of the initial surgery, the histological findings at four months after tezepelumab treatment show a marked decrease in eosinophils in the tissues.

Informed consent was obtained from the patient for the publication of this report. A 74-year-old woman with a history of adult-onset asthma, rhinitis, and hearing loss, and no history of smoking, was diagnosed with asthma at 32 years of age and prescribed a fixed-dose combination of inhaled corticosteroids (ICS)/long-acting β2-agonist (LABA). She had no history of cardiac problems. However, she experienced repeated exacerbations during winter and was hospitalized several times for asthma attacks. At the age of 55 years, she developed nasal obstruction and rhinorrhea, and at the age of 60 years, she underwent ESS at another hospital. She experienced hearing loss at 72 years of age and was diagnosed with ECRS and secretory otitis media. A respiratory physician at another hospital treated the patient for asthma with frequent use of OCSs. She had a history of a compression fracture. The patient’s score on the Japanese Epidemiological Survey of Refractory Eosinophilic Chronic Rhinosinusitis (JESREC) for ECRS diagnosis was 17. The eosinophil count from an ethmoid sinus polyp sample, obtained during surgery at another hospital, was 397/HPF (Figure 1A).

**Figure 2:**
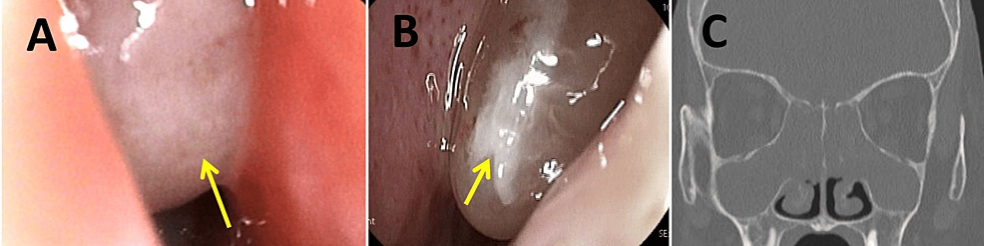
Nasal findings prior to tezepelumab treatment. (A, B) Intranasal findings. Nasal endoscopy at the time of initiation shows nasal polyps on the right side (A) and left side (B), extending from the middle nasal meatus to the inferior margin of the middle nasal concha (yellow arrow). (C) Computed tomography images before tezepelumab treatment. CT scan of the sinuses at initiation revealed soft tissue density lesions in all bilateral sinuses.

Eight months after the initial visit to our Ear, Nose, and Throat Department, the patient complained of breathlessness, and asthma treatment was initiated at our hospital's Department of Allergy and Rheumatology. Her medication was changed to ICS/long-acting muscarinic antagonist (LAMA)/LABA and then to high-dose ICS/LAMA/LABA. However, no improvement was observed. Nasal endoscopy revealed bilateral nasal polyps extending from the middle nasal meatus to the inferior margin of the middle nasal concha, with a total polyp score (TPS) of 6 (Figures 2A-2B). Computed tomography (CT) imaging of the sinuses revealed soft tissue density lesions in all bilateral sinuses, with a Lund-Mackay CT score (LMS) of 24 (Figure 2C).

**Figure 3:**
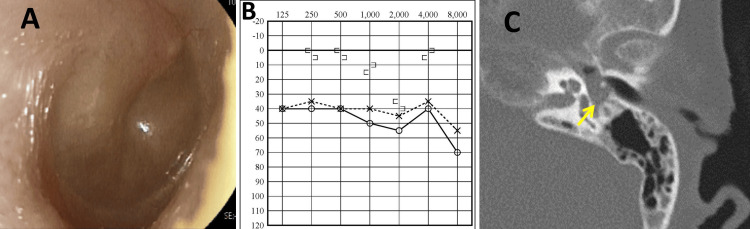
Ear findings prior to tezepelumab treatment. (A) Otoscopic findings before tezepelumab treatment: Swelling of the bilateral posterior superior quadrant of the tympanic membrane and middle ear effusions were observed. (B) Pure tone audiometry before tezepelumab treatment: Pure tone audiometry revealed a conductive hearing loss. (C) Temporal bone CT scan showing soft tissue shadows in the tympanic cavity before treatment (yellow arrow).

The proportion of blood eosinophils was 15.8%, blood eosinophil count was 1220, and total IgE levels were 179 IU/mL. The total subjective nasal and Sino-Nasal Outcome Test (SNOT)-22 score was 59. The T&T olfactometer threshold test indicated a threshold of 5.8. Bilateral swelling of the posterior superior quadrant of the tympanic membrane and middle ear effusions were observed (Figure 3A). Pure tone audiometry revealed a conductive hearing loss of 48.8 dB in the right ear and 41.3 dB in the left ear, with an air-bone gap exceeding 30 dB (Figure 3B). CT imaging showed soft tissue shadows in both the tympanic cavities and mastoid air cells (Figure 3C).

**Figure 4:**
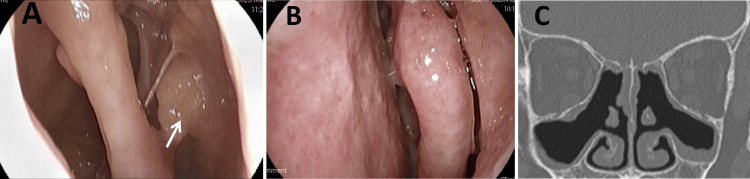
Nasal findings after four months of treatment. (A,B) Intranasal findings. (A) Right side polyps remain in the olfactory fissure on the right (white arrow), (B) On the left, the polyps have disappeared. (C) Paranasal sinus computed tomography images. All sinus shadows improved four months after treatment.

Asthma examination results showed a fractional exhaled nitric oxide (FeNO) level of 33 ppb, forced expiratory volume in 1 second (FEV1) of 0.92, %FEV1 of 57.5%, Asthma Control Test (ACT) score of 17, and Asthma Control Questionnaire 5 (ACQ5) score of 1.4. Therefore, three months after the patient's first visit to the Internal Medicine department, subcutaneous monthly doses of tezepelumab (210 mg) were initiated. We opted for tezepelumab first due to the patient's extensive clinical history and our suspicion regarding the complexity of her condition. Four months after tezepelumab initiation, the patient showed significant improvement. The total SNOT-22 score decreased to 39, TPS reduced to 3 (Figure 4A-4B), and sinus CT findings improved, with an LMS of 11 (Figure 4C).

**Figure 5:**
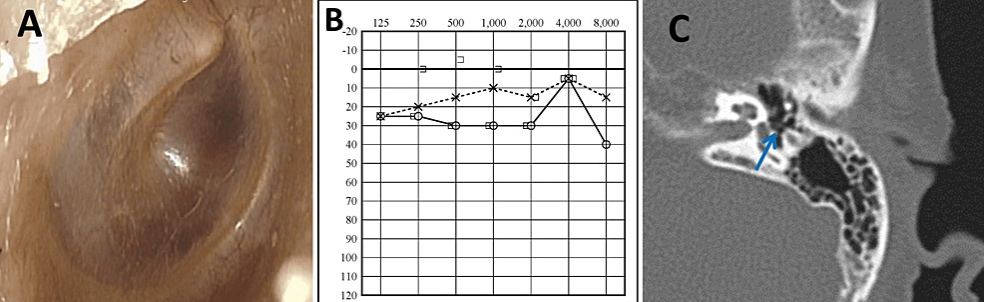
Ear findings after four months of treatment. (A) Otoscopic findings after treatment. The tympanic effusion resolved. (B) Pure tone audiometry after treatment. The air-bone gap is reduced in both ears. (C) Temporal bone CT scan. After treatment, soft tissue shadows in the bilateral tympanic cavities lightened (blue arrow).

The tympanic effusion resolved (Figure 5A). The proportion of blood eosinophils was 3.8%, the blood eosinophil count was 230, and total IgE levels were 141 IU/mL. The olfactory T&T test threshold improved to 3, and the pure tone audiometric airway threshold improved to 30.0 dB on the right and 12.5 dB on the left (Figure 5B), and soft tissue shadows in the bilateral tympanic cavities were lightened on temporal bone CT (Figure 5C).

There was a mild improvement of bronchial asthma, with an FEV1 of 1.04, %FEV1 of 65%, and a FeNO level of 13 ppb. Subjective improvement was noted as the ACT score increased to 20 and the ACQ5 score decreased to 1.2. The patient's condition was managed without OCSs. A biopsy of the remaining polyp in the right olfactory cleft, conducted three months after starting tezepelumab, revealed an eosinophil count of 17/HPF, which was a significant decrease compared to the preoperative count (Figure 1B).

## Discussion

ECRS is characterized by predominant ethmoid sinus involvement, intractable nasal polyps with eosinophilic infiltration, and comorbid bronchial asthma. According to the classification system proposed by the latest European Rhinologic Society Guidelines, ECRS is a bilateral type 2 inflammatory disease [1]. Type 2 immune reactions in CRSwNP have been associated with asthma comorbidity, severity, and recurrence of nasal polyps after systemic GCS or surgical treatment. Frequent oral GCS boosts per year and repeated surgeries are, therefore, a clear hint for type 2 CRSwNP. Other type 2 signs are the presence of comorbid late-onset asthma, the diagnosis of aspirin-exacerbated respiratory disease (AERD), histopathologic findings of eosinophils in former surgery specimens, and finally elevated blood eosinophil counts and increased polyclonal serum IgE concentrations. In Japan, ECRS was first defined by Haruna S et al. [4] in 2001. The multicenter JESREC study conducted in 2015 established ECRS diagnostic criteria based on the JESREC score [5]. The pathogenesis of ECRS involves type 2 inflammation and the release of cytokines such as IL-4, IL-13, and IL-5, which cause eosinophilic inflammation and mucin formation [6,7]. External stimuli, such as coagulolytic molds, antigens, and viruses, induce the release of epithelial cytokines (e.g., IL-33 and TSLP) from the nasal mucosal epithelium. These cytokines subsequently activate the receptors on T cells, type 2 innate lymphoid cells (ILC2s), and mast cells, leading to the production of large amounts of type 2 cytokines (IL-4, IL-5, and IL-13) [6]. Several studies have reported the efficacy of biological agents in treating type 2 inflammatory diseases. For example, dupilumab, a biological agent that blocks the shared receptor components of IL-4 and IL-13, is the only antibody product approved for treating CRSwNP in Japan. It decreases polyp size, sinus opening, and symptom severity [7]. Dupilumab, an anti-IL-4 receptor (R)alpha drug, is effective in patients with moderate-to-severe bronchial asthma, atopic dermatitis, and chronic rhinosinusitis with nasal polyps. Iino Y et al. reported that dupilumab was also effective in patients with severe refractory EOM who did not respond to treatment, including molecular-targeted therapies [2]. Nakashima D et al. investigated the efficacy of dupilumab in patients with EOM complicated by ECRS [8]. Further, both mepolizumab (anti-IL5 antibody) and benralizumab (anti-IL5 receptor antibody) have been reported to be effective against EOM [9]. Impressive reductions in CRSwNP disease burden have been documented in terms of reduction of polyp mass, sinus involvement, asthma symptoms and lung function tests, asthma control, and an increase in QOL, including work performance and absenteeism. Specifically, the ability to smell is not only of great importance for the patients but also is regained by many patients within just 4-8 weeks of treatment. It is evident that biologics can and will compete with the current 'last resort' treatment, which involves extended surgical procedures. Decisions regarding their use must carefully balance their effects, side effects, and potential complications with the active involvement of the patients. It is highly likely that future care pathways will integrate biologics with surgery or build upon systemic GCSs when managing severe, uncontrolled CRSwNP. Consequently, tezepelumab, a human monoclonal antibody inhibiting TSLP, has garnered significant attention in recent years [3]. It marks as the sixth biological agent proposed for the treatment of allergies and asthma.

TSLP is a cytokine produced by airway epithelial cells in response to inflammatory agents or mechanical stimuli [10]. TSLP activates dendritic cells and innate lymphocytes (ILC2s), induces type 2 cytokine production by Th2 cells, directly activates mast cells and basophils, and induces Th2 cytokine, IL-5, and IL-13 productions [11]. Furthermore, TSLP stimulates the differentiation of eosinophil precursor cells into mature eosinophils in airway tissues [12] and induces the release of Th17 cells, which are involved in both type 2 and non-type 2 inflammation [13]. In a previous report on bronchial asthma, TSLP expression was increased in the airways of patients with asthma and correlated with asthma severity. The inhibition of TSLP prevents asthma exacerbation and improves asthma control [9]. A multicenter randomized, double-blind, placebo-controlled trial showed that the rate of asthma exacerbation at 52 weeks was significantly lower in the tezepelumab group than in the placebo group [3]. Additionally, regardless of the baseline eosinophil count, FeNO level, or allergic status, patients with poorly controlled severe asthma treated with tezepelumab had fewer exacerbations and better lung function, asthma control, and health-related QOL than patients who received placebo. In addition, increased TSLP expression and activity have been observed in nasal polyp tissues compared to healthy sinus tissues and tissues of patients with CRSwNP [14]. This suggests that tezepelumab may also be effective in treating ECRS, a subgroup of CRSwNP. Miura et al. [15] reported that the presence of epithelial-derived TSLP in the Eustachian tube plays a crucial role in the pathogenesis of EOM. In this case, we did not perform a tympanostomy to avoid ear discharge associated with tympanic membrane perforation. EOM requires the presence of eosinophils in the middle ear effusion, which was not demonstrated in this case. Therefore, secretory otitis media did not meet the diagnostic criteria for EOM [2], thus precluding the diagnosis of EOM. The mechanism may be that the reduction of the nasal polyps may have improved the ventilation of the Eustachian tube, which may have improved the otitis media with effusion. However, it remains possible that this condition was EOM since the airborne gap was above 30 dB.

In this case, the FeNO level was relatively low under inhaled steroid treatment. However, the Global Initiative for Asthma defines type 2 inflammation as a FeNO level of 25 ppb or higher. Chest CT imaging showed no emphysematous changes in this case, and type 2 inflammation was suspected due to concomitant ECRS.

In this case, endoscopic findings of ECRS and otoscopic findings of EOM significantly improved after four months of tezepelumab treatment. Blood and tissue eosinophil counts decreased following tezepelumab treatment in this patient, whereas blood eosinophil counts often increased after dupilumab treatment. This finding suggests that tezepelumab can reduce Th2 biomarker levels upstream. Clinically, tezepelumab improved asthma symptoms, ECRS subjective symptoms, and other results, such as nasal polyp and CT scores. Tympanic and hearing test findings also improved without the use of OCSs.

Tezepelumab is considered effective regardless of biomarkers, although subanalyses have shown that it is more effective in patients with high type 2 inflammatory biomarker levels. Tezepelumab was predicted to be highly effective in this patient with CRSwNP because of high type 2 inflammation.

Although the efficacy of anti-TSLP agents for the treatment of ECRS and EOM has not been reported previously, the improvements observed with tezepelumab in this case clearly suggest that TSLP plays an essential role in the pathogenesis of ECRS and secretory otitis media associated with asthma. Further studies are required to confirm these findings and determine the long-term efficacy and safety of tezepelumab for treating ECRS and EOM.

## Conclusions

Herein, we reported a case in which tezepelumab, administered to a patient with refractory asthma, markedly improved the outcome of ECRS and secretory otitis media, both subjectively and objectively. By suppressing type 2 inflammation upstream, tezepelumab holds great promise for patients with ECRS and secretory otitis media who do not respond adequately to conventional treatment. This case did not meet the diagnostic criteria for EOM, and, as a result, its effectiveness for EOM could not be demonstrated. Further research is needed to evaluate the efficacy of tezepelumab for ECRS cases and confirmed EOM cases.
